# Evaluating the Status of Hepatitis B Vaccination in Healthcare Workers at a Central Laboratory in a Tertiary Care Hospital and Research Centre

**DOI:** 10.7759/cureus.68069

**Published:** 2024-08-28

**Authors:** Rashmi M Karigoudar, Sanjay M Wavare, Smitha O Bagali, Praveen R Shahapur, Mahesh H Karigoudar, Annapurna G Sajjan

**Affiliations:** 1 Microbiology, Shri B.M. Patil Medical College, BLDE (Deemed to be University), Vijayapura, IND; 2 Pathology, Dr Karigoudar Diagnostic Laboratory, Vijayapura, IND

**Keywords:** elfa, hepatitis b vaccination, elisa, healthcare workers, anti-hbsag titre

## Abstract

Introduction

Hepatitis B virus is a significant occupational hazard for healthcare workers worldwide. Long-term protection against hepatitis B infection is conferred by the vaccine and the protective immune response is indicated by anti-hepatitis B surface antigen (HBsAg) titre. It is crucial to monitor anti-HBsAg titres as their levels decrease over time. The study aims to evaluate the status of hepatitis B vaccination among personnel working in the Central Laboratory of Shri B.M. Patil Medical College Hospital and Research Centre. The focus is on understanding the immunization practices and protection levels against HBV within this high-risk group.

Materials and methods

A cross-sectional analysis was conducted, including collecting demographic data, determining HBsAg status, evaluating anti-HBsAg titre value, and getting vaccination details of the laboratory personnel. The study participants included doctors, lab technicians, and attendants who were assessed for both vaccination coverage and immunity levels. After obtaining their written consent, 4 ml of blood was collected in sterile blood collection tubes. All the samples were tested for HBsAg. The negative samples were tested for anti-hepatitis B surface antigen antibody (HBsAg-Ab (IgG)) titre. The enzyme-linked immunosorbent assay (ELISA) method was used to evaluate the obtained samples for HBsAg and anti-HBsAg titre.

Results

A total of 99 healthcare workers were included in the study. Most of the laboratory healthcare workers were in the age range of 20-30 years. In 84.8% of the subjects, protective antibody levels (>10 IU/ml) were found. The highest protection was seen among doctors (94.5%), followed by lab technicians (82.9%) and attendants (66.6%). However, 15.2% exhibited inadequate immunity, predominantly among the attendants (33.3%). The highest vaccination coverage was among doctors (91.8%), followed by lab technicians (78.7%) and attendants (53.3%). Most doctors had completed the full vaccination schedule (70.2%) or received a booster dose (24.3%) compared to lab technicians (57.4%) and attendants (46.6%).

Conclusion

The study highlights effective preventive measures against HBV among laboratory healthcare workers, as indicated by the absence of active infections. But it also emphasizes the necessity of focused initiatives to raise vaccination rates, particularly among attendants, in order to guarantee complete protection against HBV for all levels of laboratory workers.

## Introduction

Hepatitis B virus (HBV), which is extremely contagious, is a serious global health issue, with more than 300 million people worldwide having developed chronic HBV, accounting for about one-third of all the cases [1]. Healthcare workers are particularly exposed to blood and mucosal membranes, putting them at risk of contracting HBV infection [1]. Patients may also be at risk from healthcare workers (HCWs) who are infected with HBV because there is evidence that treating physicians may carry the virus to their patients [2]. According to the World Health Organization (WHO), 296 million people worldwide had a chronic hepatitis B infection in 2019, and 1.5 million new cases were reported annually [3]. WHO estimates that 66,000 HBV infections occur worldwide each year, affecting 5.9% of HCWs who are exposed to blood-borne HBV infections [4]. In addition to needlestick injuries, HBV can also be transmitted through cuts from contaminated sharp instruments and contact with contaminated blood or bodily fluids. This makes HBV a significant occupational hazard for HCWs worldwide [5]. Timely vaccination at 0-16 months can prevent HBV infection with a vaccine effectiveness rate of 95%. Long-term protection against hepatitis B is conferred by the vaccine, and a protective immune response is indicated by anti-HBsAg (hepatitis B surface antigen) titre [6]. It is crucial to monitor anti-HBsAg titres as their levels decrease over time [7]. Although three vaccination doses are generally sufficient to produce protective antibody levels, there has been ongoing debate about the necessity of booster doses as antibody levels decrease over time [8]. Assessing the immunological status after immunization in sensitive populations, particularly HCWs, is essential [9]. Achieving and maintaining universal vaccination coverage against HBV infection faces significant challenges in many healthcare systems, even with the availability of an effective vaccine [1].

The purpose of this study was to assess the hepatitis B vaccination status of healthcare workers in the Central Laboratory of the Shri B.M. Patil Medical College Hospital and Research Centre. The research provides important insights into current vaccination practices in this laboratory setting by evaluating vaccination coverage and detecting any immunization gaps. The results will help design focused measures to change vaccination uptake and guarantee that the laboratory workers have a strong protection against HBV. Furthermore, this research will enhance the understanding of vaccination compliance among medical professionals and facilitate the execution of efficient regulations and instructional programs to encourage all-round hepatitis B vaccination.

## Materials and methods

This cross-sectional study was conducted on healthcare workers working in the clinical pathology, microbiology, and biochemistry laboratories at the Central Laboratory of the Shri B.M. Patil Medical College Hospital and Research Centre. Informed consent was obtained from the participants prior to the study, and the subjects were provided a thorough explanation of the nature and objectives of the research. With a proportion of anti-HBsAg titre >10 IU/ml in 90.3% [10] of vaccinated people at a 95% confidence level and at 6% margin of error, the calculated sample size is 94. Therefore, the study included 99 laboratory healthcare workers. All the laboratory healthcare workers working in the Central Laboratory in the respective departments, including doctors, senior and junior residents, technicians, phlebotomists, and attendants, were included in the study.

All healthcare professionals were included in the study, regardless of their age or gender. The study excluded non-healthcare workers, healthcare workers who tested positive for HBsAg antigen, healthcare workers with a history of immunosuppressive medication, and healthcare workers with chronic liver disease.

Detailed histories of all the study participants were recorded, including name, sex, age, date of joining the Central Laboratory, and previous history of HBV vaccination (date and dose). After obtaining their written consent, 4 ml of blood was drawn and collected in sterile blood collection tubes. All the samples were tested for HBsAg by enzyme-linked immunosorbent assay (ELISA) using a kit (HBsAg ultra kit manufactured by bioMérieux, Marcy-l'Étoile, France) according to the manufacturer’s protocol. The negative samples were tested for anti-HBsAg-Ab (IgG) titre using the VIDAS-PC (bioMérieux) Anti-HBs assay, which operates on the principle of the enzyme-linked fluorescent assay (ELFA). This method combines a two-step enzyme immunoassay sandwich procedure with fluorescence detection. The VIDAS-PC system is fully automated and utilizes single-dose ready-to-use reagents [11]. Statistical analyses were performed using SPSS Version 20 (IBM SPSS, Armonk, NY). The demographic characteristics, vaccination status, and anti-HBsAg titres of healthcare workers were summarized using frequency and percentage distributions. The chi-square test was employed to examine associations between categorical variables, such as vaccination status across different occupations and anti-HBsAg titre values relative to vaccination status and occupation. A p-value of less than 0.05 was considered statistically significant for evaluating the strength of these associations.

## Results

A total of 99 healthcare workers were included from the Central Laboratory of Shri B.M. Patil Medical College Hospital and Research Centre in Vijayapura over a period of one year. The study population consisted of 37 doctors, 47 lab technicians, and 15 lab attendants. The maximum number of participants (36) were in the 20- to 30-year age range, and the minimum (19) were in the 40- to 50-year age group (Table 1).

**Table 1:** Age-wise distribution of healthcare workers

Age (years)	Number of participants
20-30	36
30-40	24
40-50	19
50-60	20
Total	99

Female preponderance was observed among doctors and attendants, while male preponderance was seen among lab technicians. Positive anti-HBsAg titres (>10IU/ml), indicating immunity, were found in 84 participants, while 15 subjects showed negative titres (<10IU/ml). Among those with positive titres, 94.5% were doctors, 82.9% were lab technicians, and 66.6% were attendants. Negative titres were observed in 5.4% of doctors, 17.02% of lab technicians, and 33.3% of attendants (Table 2).

**Table 2:** Anti-HBsAg titre in healthcare workers Chi-square value=6.719; p=0.0348

Anti-HBsAg titre	Doctors		Technicians		Attenders		Total
	No	%	No	%	No	%	
Positive (>10 IU/ml)	35	94.5	39	82.9	10	66.6	84
Negative (<10 IU/ml)	2	5.4	8	17.02	5	33.3	15
Total	37		47		15		99

Among the healthcare workers, most of the doctors (91.8%) were vaccinated against hepatitis B compared to lab technicians (78.7%) and attenders (53.3%). Among doctors, eight had taken only a single dose, 17 had completed three doses of vaccine but had not taken the booster dose. While nine had completed three doses of vaccine with a booster dose, only three were not vaccinated. Among the lab technicians, seven had a single dose, 27 had completed the full three doses of vaccine and none had taken a booster dose, while 13 were not vaccinated. Among the attenders, one had a single dose, six had completed the schedule without a booster dose, one had taken complete three doses of vaccine along with a booster dose, and seven were not vaccinated. Vaccination status was significantly higher among the doctors compared to laboratory technicians and attenders (Table 3).

**Table 3:** Vaccination status of healthcare workers Chi-square value=8.7740; p=0.0124

Vaccination Status	Doctors		Lab Technicians		Attenders		Total	
	No	%	No	%	No	%	No	%
Single dose	8	21.62	7	14.89	1	6.67	16	16.16
Complete three doses (without booster dose)	17	45.95	27	57.45	6	40.00	50	50.51
Complete three doses (with booster dose)	9	24.32	0	0	1	6.67	10	10.10
Not vaccinated	3	8.11	13	27.66	7	46.67	23	23.23
Total	37		47		15		99	

Among the doctors, nine who were immunized with a booster dose showed positive titres, 17 who were immunized without a booster dose showed positive titres, eight who were partially immunized showed positive titres, and three who were not immunized showed negative titres. Among the lab technicians, 27 were immunized without a booster dose, of which 26 showed positive titres and one negative titre. Seven who were partially immunized showed positive titres, and out of the 13 who were not immunized, six showed negative titres and seven showed positive titres (Table 4).

**Table 4:** Anti-HBsAg titre levels and immunization status of healthcare workers Chi-square value=40.870; p<0.0001

Anti-HBsAg titre	Doctors		Lab Technicians		Attenders	
	<10I U/ML (negative)	>10I U/ML (positive)	<10 IU/ML (negative)	>10 IU/ML (positive)	<10 IU/ML (negative)	>10 IU/ML (positive)
Immunized with a booster dose	0	9	0	0	0	1
Immunized without a booster dose	0	17	1	26	0	6
Partially immunized	0	8	0	7	0	1
Non-immunized	2	0	6	7	5	2

## Discussion

The risk of acquiring hepatitis B infection increases for healthcare workers due to their increased exposure to various bodily fluids [5]. In comparison to the general public, the risk of contracting HBV infection among healthcare workers is three to five times higher. Estimating the percentage of healthcare professionals who have received the hepatitis B vaccine is necessary to determine their susceptibility to HBV infection by detecting anti-HBsAg titre levels [12]. Assessing the hepatitis B vaccination status of healthcare workers offers a vital understanding of the immunization protocols and protection against HBV infection within this high-risk population. 

The age distribution indicates that most laboratory healthcare workers are in the younger age range of 20-30 years (36%), followed by 30-40 years (24%). This trend aligns with the typical age profile of healthcare workers, where a substantial proportion are often early in their careers. Similar findings were reported in a study by Singhal et al., which showed a predominant younger age group among healthcare workers in Indian healthcare settings [13]. The gender distribution in the study shows a higher prevalence of males among lab technicians (80.8%), while females dominate among doctors (70.2%) and attendants (60%).

The absence of HBsAg-positive cases among all study participants is a positive finding, indicating no active HBV infection within the laboratory personnel at the time of the study. This result suggests that the existing preventive measures, including vaccination and other infection control practices, are effective in preventing HBV transmission among these workers.

The vaccination status reveals that a significant proportion of the personnel were immunized: 91.8% of doctors, 78.7% of lab technicians, and 53.3% of attendants. In a study conducted by Batra et al., vaccination coverage among various healthcare professionals was reported as follows: 92.4% among doctors, 62.4% among medical students, 41.7% among nursing staff, 24.2% among laboratory technicians, and 12.1% among nursing students, while none of the laundry staff were vaccinated [14]. Among the various groups of vaccinated healthcare workers, the vaccination rate was highest among nursing staff (74.9%), followed by doctors (13.8%), housekeeping staff (4.9%), laboratory technicians (4.5%), and administrative staff (2%) [10].

This data underscores the relatively high vaccination coverage among doctors, which could be due to better health literacy and easier access to vaccination programs. The lower immunization rate among attendants highlights the need for targeted interventions to improve vaccine uptake in this group. Further analysis of the vaccination details shows that most doctors had either completed the full vaccination schedule (70.2%) or received a booster dose (24.3%). Among the lab technicians, 57.4% had completed the full dose, while none had received a booster dose. Among the attendants, only 46.6% had been vaccinated, with the majority having either not received the full dose or not been vaccinated at all. According to a related study conducted in New Delhi, 55.4% of healthcare workers had received all recommended vaccinations against HBV [15]. A somewhat lower incidence of fully vaccinated HCWs (42.2%) was found by Kumar et al. [16].

In the present study, the analysis of anti-HBsAg titres revealed that 84.8% of the participants had protective antibody levels (>10 IU/ml), indicating successful immunization, and resulting immunity against HBV. The highest percentage of positive titres were detected in doctors (94.5%), followed by lab technicians (82.9%) and attendants (66.6%). This gradient may reflect differences in vaccination uptake and possibly occupational health practices among the different categories of personnel. These findings are similar to a study by Jha et al., which reported similar protective antibody levels among healthcare workers in Nepal. Conversely, 15.2% of the participants had negative anti-HBsAg titres, indicating inadequate immunity. This group includes a higher proportion of attendants (33.3%) compared to technicians (17.02%) and doctors (5.4%) [10]. The lower immunity levels among attendants could be attributed to several factors, including lower vaccination rates, differences in access to healthcare services, or varying levels of awareness about the significance of vaccination [6]. In another research, 30% of the vaccinated healthcare workers, primarily doctors and medical students, had anti-HBs titre values less than 10 IU/mL, while the remaining 70% had protective titres greater than 10 IU/mL. This indicated that 30% of HCWs who had received vaccinations were still susceptible to contracting HBV [14]. In contrast, in another study, the anti-HBs titre was less than 10 IU/ml in 2.9% of doctors and 12.4% of nurses, whereas it was greater than 10 IU/ml in 97.05% of the doctors and 87.56% of the nurses. This demonstrated that 12.4% of the nurses and about 3% of the doctors are still susceptible to contracting HBV [15]. According to a related study by Batra et al., 30% of the individuals were at risk of HBV infection [14]. Our results were in line with those of previous studies by Alimonos et al. [17] and Zeeshan et al. [18], which reported good antibody response rates of 92% and 86%, respectively.

Most individuals who received the entire vaccination schedule without a booster dose had positive antibody titres, according to the thorough analysis comparing vaccination status with anti-HBsAg titres. Positive titres were seen in a small percentage of partially and non-immunized people nevertheless, which may indicate prior HBV exposure and the establishment of natural immunity. These data collectively demonstrate the urgent need for further initiatives to raise vaccination rates, especially among lower-level medical staff and to guarantee that booster shots are given when necessary to preserve protection. Protecting all healthcare workers from HBV infection requires boosting vaccination programs, enhancing access to healthcare services, and raising awareness of the significance of HBV immunization. The fact that all doctors who got booster doses had positive titres, supports the idea that these doses are useful for preserving long-term immunity. High levels of immunity were also shown by lab personnel who finished the immunization regimen, but the lack of booster shots raises questions about how long-lasting this protection will be. The attendees had the lowest positive titre levels, which highlights the need for comprehensive immunization measures and reflects the lower overall vaccination coverage. This research draws attention to the high vaccination rates among physicians, which is indicative of successful immunization programs within this population. Effective preventive measures are evident as no active HBV infection was found among the participants.

Limitations of the study

The study included only 99 healthcare workers, which limits how far the findings may be extended to different situations or populations. The cross-sectional nature of the study captures the vaccination status and antibody titres at a single point in time. Neither the participants' long-term immunity nor the potential need for booster dosages is tracked after the trial period. While the study assessed anti-HBs titres to determine their immunity levels, it did not evaluate other aspects of vaccine efficacy or potential factors influencing immune responses, such as previous HBV exposure or genetic factors.

## Conclusions

This study found that 91.8% of the doctors, 78.7% of the lab technicians, and 53.3% of the attendants were vaccinated. The lower vaccination coverage among attendants is particularly concerning and suggests a need for targeted intervention. Protective antibody levels (>10 IU/ml) were observed in 94.5% of the doctors, 82.9% of the lab technicians, and 66.6% of the attendants. This reflects generally effective immunization but also reveals inadequate immunity in 15.2% of the participants, with the highest rate of insufficient immunity among the attendants (33.3%). Among the doctors, 24.3% received a booster dose, which is associated with higher immunity levels. None of the lab technicians and only 6.67% of the attendants received a booster, highlighting a critical area for improvement. It is necessary to implement improved educational initiatives, expand access to vaccination services, and strengthen occupational health policies. Through these measures, healthcare institutions can improve vaccination coverage and ensure robust protection against HBV infection for all healthcare workers. This approach will contribute to a safer working environment and better overall health outcomes for healthcare staff.
